# Developing the Next Generation of Leaders in Health Policy and Management: Lessons From an Undergraduate Student-Led Organization

**DOI:** 10.3389/fpubh.2022.855712

**Published:** 2022-03-18

**Authors:** Charlotte Thomas, Sahil Sandhu, Josee Li, Gillian D. Sanders, Janet Prvu Bettger

**Affiliations:** ^1^Trinity College of Arts & Sciences, Duke University, Durham, NC, United States; ^2^Harvard Medical School, Boston, MA, United States; ^3^Duke-Margolis Center for Health Policy, Duke University, Durham, NC, United States; ^4^Department of Population Health Sciences, Duke University School of Medicine, Durham, NC, United States; ^5^Duke Clinical Research Institute, Duke University School of Medicine, Durham, NC, United States; ^6^Department of Orthopaedic Surgery, Duke University School of Medicine, Durham, NC, United States

**Keywords:** education, undergraduate (MeSH), health policy, healthcare, management-healthcare, student organization

## Abstract

As health care continues to evolve, training the next generation of healthcare leaders is more important than ever. However, many university undergraduate students are not directly exposed to topics such as health policy and management within their coursework or co-curricular engagements. At Duke University, we developed the Student Collaborative on Health Policy (SCOHP) as an inter-disciplinary health policy hub that offers opportunities for learning, engagement, and leadership in the healthcare-related fields for students of all academic backgrounds. We see opportunity for similar student-led groups to be established by student leaders at other institutions, increasing interaction with experts, mentorship and the accessibility of experiential education, service, and leadership in the health care sector.

## Introduction

Tackling tomorrow's health system challenges will require a workforce equipped with the tools to improve healthcare value and equity. With a national commitment to care re-design and payment reform, the next generation of healthcare leaders will need to work across sectors and professions, including clinical care, administration, public service, research, and industry. Universities can play a critical role in developing the pipeline of healthcare leaders, as early as at the undergraduate level.

Unfortunately, few universities offer undergraduate students adequate exposure to health policy and management through curricular and co-curricular programming. While some institutions include a health policy and management track within public health majors (1), these programs may only reach a small percentage of students interested in healthcare careers. There is a need for scalable and sustainable models to engage undergraduate students across degree programs.

One model for engagement is student organizations, which are effective outlets for students to develop leadership and professional skills, serve their local communities, and develop a supportive network of peers with similar interests (2–4). At Duke University, we developed the Student Collaborative on Health Policy (SCOHP) in partnership with the Duke-Margolis Center for Health Policy. SCOHP's mission is “to unite students across disciplines and the University in a collaborative effort to increase awareness and opportunities in health policy” (5).

In this piece, we describe our process for developing our student organization's structure and opportunities for engagement. Our model and lessons learned can be a resource for students and health policy experts at other universities to partner to create their own student-led organizations or enhance existing graduate student organizations' (e.g., AcademyHealth student chapters, medical student advocacy initiatives) reach or include the undergraduate population. It is critical that awareness and access to opportunities in health policy begin earlier in the pipeline of preparing future leaders in all the facets of health and health care.

## Crafting a Student-Organization Structure

The Duke-Margolis Center for Health Policy was established in 2016. Undergraduate students recognized a gap in the Center's strategic plan and established a student advisory committee to assess needs and co-design extracurricular opportunities in health policy and management with Center leadership. As engagement increased, students recommended establishing a formal organization. SCOHP was subsequently launched after a planning period in the 2018–2019 academic year. To become an official university-recognized student organization and eligible for funding support, we applied to our university's student government in 2019 (e.g., written constitution, proof of student interest and faculty advisor). After receiving approval, we advertised SCOHP to the student body via email, social media, and targeted presentations in healthcare-related classes. Interested students were invited to apply to join our executive board and attend a kick-off meeting, where students participated in a series of brainstorming activities to share their own interests to guide future projects.

**Figure 1:**
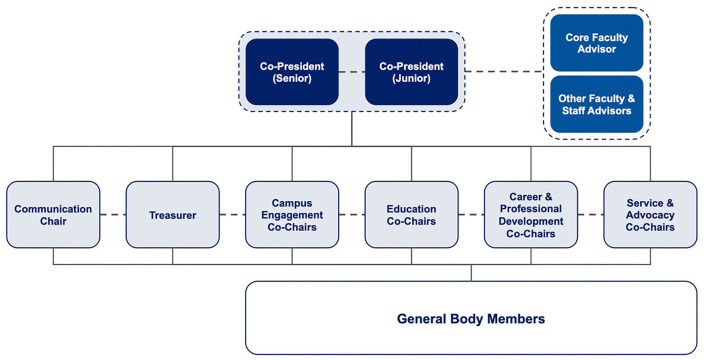
Structure of student organziation.

SCOHP's organizational structure establishes several levels of leadership and advising structured around SCOHP's strategic priorities: campus engagement, education, career and professional development, service and advocacy (Figure 1). Leading the organization are two co-presidents (a primary president and a secondary president), who oversee the executive board. The executive board meets biweekly and consists of the co-presidents, treasurer, communications director, and co-chairs of committees. Each committee is led by two co-chairs, meets weekly or bi-weekly with a broader group of general body members, and carries out a variety of educational and service activities. The entire general body meets monthly for each committee to share updates. The executive board also meets monthly with a faculty and staff advisory committee from the Duke-Margolis Center to identify opportunities for collaborative efforts between SCOHP, Duke-Margolis, and the local community.

## Developing Opportunities for Student Engagement

We organized SCOHP activities and projects into four committees, one for each of our four priorities (Table 1). Within each committee, we designed four levels of engagement to ensure students had different points of access based on their interests, time available, and personal or professional goals. The SCOHP committee levels of engagement are: (1) learning about health policy and management, (2) developing real-world skills, (3) applying gained knowledge and skills through service, and (4) leading their peers.

**Table 1:** Examples of 2019–2021 Projects.

**Project**	**Committee**	**Description**	**Reach**	Level of engagement*
Health Policy Week	Campus Engagement	SCOHP organized and implemented a full week of programming to increase the student body's awareness of health policy issues. Topics included health disparities, environment and health, global reproductive health, and health policy in the 2020 election.	9 students partnered with 5 other student organizations to host two live events, disseminate a podcast, and publish an infographic.	1
Podcast	Campus Engagement	SCOHP members established a student-led health policy podcast, *The Scope*. Student broadcast journalists were encouraged to follow their interests in health policy, resulting in episodes covering the intersection of health care and artificial intelligence, state policy, child policy, environmental policy, and racial justice.	12 students encompassed the broadcast journalist team and released 18+ podcasts available on multiple platforms	1
House Courses	Education	Semester-long, for-credit seminars taught by undergraduates that meet weekly. Sponsored by Duke-Margolis faculty, courses include “Transforming the US Health Care System,” “Health Care: A Human Right?,” “Health Behind Bars,” and “Drug Development for Essential Medicines.”	152 undergraduate students enrolled in 12 SCOHP house course sections across the 2019–2020 and 2020–21 academic years.	1
Curricular Design	Education	SCOHP members partnered with Duke-Margolis faculty to explore feasibility of and undergraduate interest in new health policy curricular opportunities. This included for first-years (e.g., seminar courses, weekly faculty dinners, shared housing), and a health policy certificate program, as a pipeline to Duke-Margolis's existing undergraduate scholars program and health policy courses.	8 students led proposals provided to faculty for evaluation within academic programming considerations. Proposals were submitted to Duke University administration in fall 2021.	2
Case Competition	Career and Professional Development	SCOHP members established the first annual Health Policy Case Competition for undergraduates at Duke University. Competing students utilized business problem-solving to present policy solutions to real healthcare issues. Project leaders recruited sponsors, judges, and team mentors; wrote the case (i.e., COVID-19 vaccine equity in North Carolina); and managed event logistics.	6 students planned the case competition, to which 99 undergraduates applied and 50 participated.	2
Career Guide	Career and Professional Development	In partnership with Duke-Margolis faculty and Duke alumni, SCOHP members developed a comprehensive career guide for undergraduates seeking careers in health policy and management.	6 students developed the career guide, which was distributed widely to SCOHP and the Duke undergraduate student body.	1
Help Desk Initiative	Service and Advocacy	SCOHP members launched Help Desk volunteer program to train students as community resource navigators to address patients' social needs, such as food insecurity and housing instability, at a local Federally Qualified Health Center (6).	32 students trained as community resource navigators in summer 2020 and spring 2021 cohorts, serving 500+ patients.	3
Affordable Care Act (ACA) Navigators	Service and Advocacy	SCOHP members launched student volunteer ACA Navigators program, in partnership with Legal Aid of North Carolina, to help North Carolinians enroll in healthcare coverage (7).	34 students became certified ACA Navigators, serving community members at 25 partner sites.	3
COVID-19 Resource Directory	Service and Advocacy	During the COVID-19 pandemic, SCOHP members created and maintained a county-level directory of community resources for health and social needs (8). SCOHP partnered with the county public health department, local non-profits, and health systems to maximize reach and tailor efforts to meet local needs (9).	31 student volunteers curated directory of 370+ resources.	3

*1 = learning about health policy and management; 2 = developing real-world skills; 3 = applying gained knowledge and skills through service.

First, students interested in gaining exposure to health policy can participate in SCOHP's Health Policy Week (hosted by our campus engagement committee in the fall semester), in which undergraduates engage with a different healthcare topic each day through guest speakers, forum discussions, and online resources. For a longitudinal learning experience, undergraduates looking to learn the basics about healthcare delivery and reform can enroll in a semester-long, for-credit “House Course,” taught by members of our education committee under the mentorship of Duke-Margolis core and affiliate faculty. SCOHP organizes two to four House Courses each fall and spring semester and the registered students ranging from 7 to 18 per seminar meet once a week (online since spring 2020).

Second, students hoping to develop skills in health policy and management through experiential learning could compete in our annual case competition. Planned by our career and professional development committee, and sponsored by internal and external partner organizations, the case competition allows undergraduates to develop and pitch proposed solutions for real-world health challenges to a multi-sector panel of community member and faculty judges. Participating students also gain the opportunity to network with competition sponsors and explore opportunities to interview or intern with them.

Third, our service and advocacy committee provides students with the opportunity to apply knowledge and skills in health policy through service activities and academic-community partnerships. For example, in collaboration with a non-profit law firm, we recruited and trained students as Affordable Care Act Navigators to help community members enroll in health insurance (7).

Fourth, across all four committees and the executive board, students can develop leadership skills essential for successful careers in health policy and management. Students leading committees and projects learn to translate their ideas into action, organize and motivate their peers, and mentor the next generation of students to sustain their projects beyond their own SCOHP tenure. For example, in SCOHP's second year, students decided to launch a health policy and management podcast, “The Scope.” Students developed skills in outlining 20-min episodes and developing scripts, networking with subject matter experts, recording and editing podcasts, and creating handbooks to support their peers in leading their own episodes.

## Lessons Learned and Recommendations for Adopting Our Model

Based upon our experience of developing and implementing the SCOHP model, we have learned three primary lessons that may be useful for students and health policy educators to consider when adopting our model or adapting specific elements to an existing context.

### Leveraging Existing Faculty and University Support

Essential to SCOHP's launch was faculty and staff mentorship through the Duke-Margolis Center. Our mentors fostered SCOHP's growth by collaborating on programming and facilitating academic-community partnerships for service activities. For example, our mentors' expertise was instrumental to developing a health policy career guide that best reflected the diverse career paths in the field. Our mutually-beneficial partnership also enabled the Duke-Margolis Center to expand its presence among undergraduates and have a direct connection to a large body of students with whom to share research and education opportunities.

Transitioning from an advisory committee to an official student organization allowed us to access University funds to support events targeting undergraduates, such as Health Policy Week, and to increase recruitment through student organization activity fairs and websites. We also leveraged our status as an official student organization to partner with more well-established student organizations (e.g., consulting clubs, politics and policy groups, and departmental organizations) to increase reach of SCOHP's programming. Students from other institutions with limited access to University funds should consider utilizing external resources. For example, our policy case competition has external sponsors and the AcademyHealth student chapters can apply for mini-grants and discounted conference registrations (8). Across approaches, we encourage students and faculty to be transparent about their goals and identify projects with most potential for synergy.

### Fostering a Tiered, Mentorship Model to Ensure Organization Sustainability

Maintaining an organization with 100+ members requires thoughtful mechanisms to ensure progress and sustainability. First, our committee structure allows committees to implement several projects simultaneously and independently. Nonetheless, regular check-ins with a broader general body and executive board still create a culture of accountability and peer support. We also learned that structuring primary and secondary co-positions for all leadership levels facilitates peer mentorship and smooth transfers of leadership as students graduate or explore other roles within SCOHP. When starting an undergraduate student organization, we recommended co-presidents and co-chairs represent different years and that founders include 1st and 2nd year students in their executive board. Such an approach, coupled with mechanisms for knowledge sharing and documentation (e.g., archiving meeting notes and organizational materials in a shared Google Drive, creating best practices one-pagers for programs) enables the organization to sustain and scale after the founding team graduates.

### Allowing for Multiple Levels and New Forms of Student Engagement

In our experience, students join SCOHP with varying levels of interest in health policy and engagement in the student organization. Creating low-touch (e.g., attending speaker events during our Health Policy week) and high-touch (e.g., leading and coordinating our annual case competition) opportunities for engagement can allow students to tailor their participation to best meet their needs. Inviting students to initiate projects that forge connections between health policy and their own academic and professional interests (e.g., climate change, mental health, racial justice) has been crucial for retaining students. Additionally, faculty advisors can help students identify geographically relevant health policy projects. For example, many of our service projects relate to addressing health-related social needs, given North Carolina's recent push to integrate health and social services through Medicaid reform (10). Several research projects are connected with global health priorities. Regardless of how project ideas are sourced, we recommended club leadership consider how to produce “value-add” initiatives that do not duplicate existing curricular or extra-curricular offerings.

## Conclusion

Our experience with SCOHP provides a model for undergraduate student engagement in health policy and management that other universities can replicate and adapt in their local contexts and for students at any or all learning levels. We encourage students and educators to consider partnering to create meaningful opportunities to inspire the next generation of health care and health policy leaders.

## Data Availability Statement

The original contributions presented in the study are included in the article/supplementary material, further inquiries can be directed to the corresponding author/s.

## Author Contributions

CT, SS, and JL drafted the manuscript. GS and JP revised the manuscript critically for important intellectual content and all authors approved of the version of the manuscript to be published. All authors were involved in the conception and implementation of the project.

## Conflict of Interest

The authors declare that the research was conducted in the absence of any commercial or financial relationships that could be construed as a potential conflict of interest.

## Publisher's Note

All claims expressed in this article are solely those of the authors and do not necessarily represent those of their affiliated organizations, or those of the publisher, the editors and the reviewers. Any product that may be evaluated in this article, or claim that may be made by its manufacturer, is not guaranteed or endorsed by the publisher.

## References

[1] Tarasenko YN Lee JM. U.S. Undergraduate education in public health: hot or not? Front Public Health. (2015) 3:71. 10.3389/fpubh.2015.00071 26029686 PMC4426683

[2] Smith LJ Chenoweth JD. The contributions of student organization involvement to students' self-assessments of their leadership traits and relational behaviors. Am J Bus Educ. (2015) 8:279–88. 10.19030/ajbe.v8i4.9422

[3] Zeeman JM Bush AA Cox WC Buhlinger K McLaughlin JE. Identifying and mapping skill development opportunities through pharmacy student organization involvement. AJPE. (2019) 83:6950. 10.5688/ajpe6950 31223160 PMC6581355

[4] Soria K Troisi J Stebleton M. Reaching out, connecting within: community service and sense of belonging among college students. High Educ Rev. (2012). 9:65–8.

[5] Duke Student Collaborative on Health Policy. https://sites.duke.edu/dukescohp/ (accessed June 5, 2021)

[6] Sandhu S Xu J Blanchard L. A community resource navigator model: utilizing student volunteers to integrate health and social care in a community health center setting. Int J Integr Care. (2021) 21:2. 10.5334/ijic.5501 33597833 PMC7863845

[7] Hallur S Sandhu S Herold E. Embedding student volunteer affordable care act navigators in a primary care clinic. Ann Fam Med. (2022). 10.1370/afm.2794. [Epub ahead of print]. PMC9199042 35318225

[8] Student Chapter Mini Grants. AcademyHealth. https://academyhealth.org/page/student-chapter-mini-grants (accessed December 31, 2021).

[9] Sandhu S Lemmon ME Eisenson H Crowder C Bettger JP. Addressing the social determinants of health during the COVID-19 pandemic: ensuring equity, quality, and sustainability. Fam Community Health. (2021) 44:78–80. 10.1097/FCH.0000000000000290 33351516

[10] Wortman Z Tilson EC Cohen MK. Buying health for North Carolinians: addressing nonmedical drivers of health at scale. Health Aff. (2020) 39:649–54. 10.1377/hlthaff.2019.01583 32250668

